# Expanding the Gastrointestinal Phenotype of 10p15.3 Microdeletion Syndrome: Refractory Atypical Gastroparesis in an Adult

**DOI:** 10.7759/cureus.77555

**Published:** 2025-01-16

**Authors:** Jeffrey Li, Vismaya Bachu, Bita Shahrvini, Mark Baniqued, Shida Haghighat, Niharika R Mallepally

**Affiliations:** 1 Internal Medicine, University of California Los Angeles David Geffen School of Medicine, Los Angeles, USA; 2 Internal Medicine, Olive View University of California Los Angeles Medical Center, Los Angeles, USA; 3 Vatche and Tamar Manoukian Division of Digestive Diseases, University of California Los Angeles David Geffen School of Medicine, Los Angeles, USA

**Keywords:** 10p15.3 microdeletion syndrome, atypical gastroparesis, dip2c gene, enteric nervous system dysfunction, gastroesophageal reflux disease (gerd), gastrointestinal motility disorders, prucalopride, rare genetic disorders, refractory gastrointestinal disease, zmynd11 gene

## Abstract

10p15.3 microdeletion syndrome is a rare genetic disorder characterized by the loss of *ZMYND11* and *DIP2C *genes, resulting in a range of neurodevelopmental delays, dysmorphic features, and gastrointestinal (GI) symptoms. The syndrome has been primarily reported in pediatric patients, and GI manifestations remain poorly studied - particularly in adults - given the limited number of reported cases. To date, only gastroesophageal reflux disease (GERD) and eosinophilic esophagitis (EOE) have been reported in adult patients. We present the first documented case of refractory atypical gastroparesis in a 32-year-old female with known 10p15.3 microdeletion syndrome. This case expands the GI phenotype associated with this rare syndrome and highlights the importance of recognizing motility disorders in patients with neurodevelopmental delays. Further studies are needed to explore the prevalence and underlying mechanisms of atypical gastroparesis in 10p15.3 microdeletion syndrome.

## Introduction

Rare genetic disorders, such as 10p15.3 microdeletion syndrome, often have atypical clinical presentations, posing diagnostic and management challenges. 10p15.3 microdeletion syndrome is characterized by a broad spectrum of clinical manifestations, including neurologic and developmental delays, craniofacial abnormalities, and gastrointestinal (GI) complications [1,2]. First described by DeScipio et al. in 2012 through chromosomal microarray mapping, the syndrome involves the deletion of *ZMYND11* and *DIP2C* genes on the short arm of chromosome 10 [1]. These genes play critical roles in nervous system development and regulation, and their haploinsufficiency underlies the disorder’s clinical presentation [1,3,4].

Most cases present in infancy or early childhood with intellectual disability and seizures, which often overshadow less frequent but clinically significant GI manifestations [1]. While feeding difficulties and gastroesophageal reflux disease (GERD) have been noted in some pediatric patients, GI symptoms are poorly characterized in adults. In DeScipio et al.’s seminal cohort of 19 cases, only three pediatric patients exhibited GI complications, and the two adult cases studied had no GI symptoms [1]. A single separate case described an adult patient with eosinophilic esophagitis (EOE) and GERD [5].

The rarity of 10p15.3 microdeletion syndrome and the predominance of pediatric-focused literature make the diagnosis and management of GI symptoms in adults particularly challenging. To our knowledge, there are no documented cases of motility disorders in adult patients with 10p15.3 microdeletion syndrome. We present a novel case of refractory atypical gastroparesis in an adult with known 10p15.3 microdeletion syndrome. This case was previously presented as an abstract at the 2024 American College of Gastroenterology Annual Scientific Meeting on October 27, 2024.

## Case presentation

A 32-year-old female with 10p15.3 microdeletion syndrome, intellectual disability, and well-controlled seizures presented to the GI clinic with a 1.5-year history of daily nausea and non-bilious emesis. Initially mild and intermittent, her symptoms progressively worsened over two weeks with the onset of retrosternal chest pain and epigastric abdominal pain, prompting admission at an outside hospital.

During her prior hospitalization, a cardiology workup of electrocardiogram and transthoracic echocardiogram was unremarkable, and her chest pain resolved spontaneously. Gastroenterology was consulted for persistent emesis and nausea. Laboratory studies, including complete blood count, comprehensive metabolic panel, erythrocyte sedimentation rate, and C-reactive protein were unremarkable. Tissue transglutaminase antibody and H. pylori stool antigen tests were negative. Imaging studies, including a hepatobiliary iminodiacetic acid (HIDA) scan, abdominal ultrasound, and esophagogastroduodenoscopy (EGD) with biopsies revealed no abnormalities. However, gastric emptying scintigraphy demonstrated a half-emptying time of 725 minutes (normal: <90 minutes), consistent with delayed gastric emptying (Table 1). She was discharged on metoclopramide 10 mg three times daily.

**Table 1:** Pertinent laboratory and imaging results in an adult patient with 10p15.3 microdeletion syndrome and chronic, persistent nausea and emesis HIDA: Hepatobiliary iminodiacetic acid; EGD: Esophagogastroduodenoscopy

	Results	Reference range
Pertinent lab data		
White blood cell count	5.9 x 10^3^/µL	4.0 – 11.0 x 10^3^/µL
Hemoglobin	12.6 g/dL	12 – 16 g/dL
Erythrocyte sedimentation rate	<20 mm/hr	<20 mm/hr
C-reactive protein	<5 mg/L	<5 mg/L
Tissue transglutaminase antibody	Negative	Negative
H. pylori stool antigen test	Negative	Negative
Imaging and procedures		
Abdominal ultrasound	Normal	No evidence of gallstones, masses, or hepatobiliary pathology
HIDA scan	Normal	Normal gallbladder contraction and bile flow
EGD	Normal	No esophagitis, gastritis, ulcers, or structural abnormalities
48-hour intraesophageal pH study	Normal	pH <4 in distal esophagus <4–6% of the time
Gastric emptying scintigraphy	T_1/2_ = 725 minutes	T_1/2_ < 90 minutes
Magnetic resonance enterography	Normal	No small bowel wall thickening, edema, or strictures

At her post-discharge follow-up clinic visit, the patient reported continued daily emesis and nausea despite treatment with metoclopramide. Ondansetron provided minimal relief, and omeprazole - trialed inconsistently - did not improve symptoms. She was subsequently referred to the GI clinic for further evaluation.

On initial presentation to the GI clinic, she reported nightly emesis, chest discomfort, and increased eructation. She denied dysphagia, odynophagia, diarrhea, or overt gastrointestinal bleeding. On physical examination, her abdomen was soft, non-tender, and non-distended. Given the known association between 10p15.3 microdeletion syndrome and GERD, and her prior inconsistent use of omeprazole, undertreated GERD was suspected. She was started on omeprazole 20 mg daily, titrated to 40 mg twice daily over three months, with adjunctive famotidine. Despite escalating therapy, her symptoms remained unchanged.

**Figure 1:**
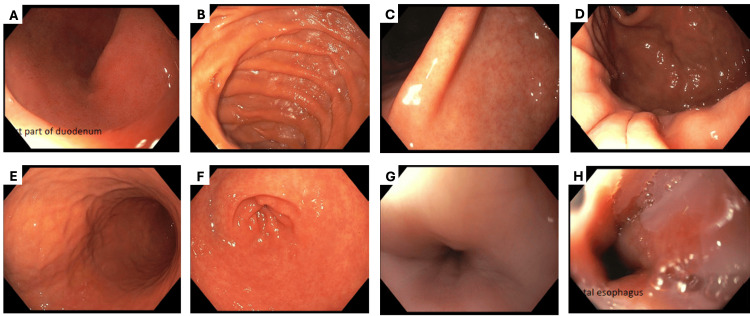
Normal repeat EGD findings Unremarkable findings on repeat EGD in D1 (duodenal bulb) (A), D2 (second portion of the duodenum) (B), angularis (C), fundus (D), body of stomach (E), antrum (F), proximal esophagus (G), and distal esophagus (H). EGD: Esophagogastroduodenoscopy

Repeat EGD confirmed normal findings, with no evidence of esophagitis, gastritis, or duodenal pathology (Figure 1). A 96-hour intra-esophageal pH study was negative for pathologic GERD, leading to discontinuation of proton pump inhibitor (PPI) therapy. Magnetic resonance enterography demonstrated no evidence of small bowel inflammation. Given her prior abnormal gastric scintigraphy study and non-response to metoclopramide, prucalopride 1 mg daily was started for refractory gastroparesis.

Over the next two months, the patient demonstrated significant clinical improvement. Her emesis frequency decreased from daily to 1-2 episodes per week, appetite and food tolerance improved, and she gained 10 pounds. At her most recent follow-up, she continued to report stable symptoms and improved quality of life while tolerating prucalopride without adverse effects.

## Discussion

Recognition and management of GI disease in adult patients with rare genetic disorders, such as 10p15.3 microdeletion syndrome, is nuanced. A comprehensive literature review revealed a limited characterization of GI symptoms associated with this condition. In neonates, early feeding difficulties - often related to craniofacial abnormalities causing dysphagia - are the most commonly cited GI manifestations, occurring in 12% of patients in DeScipio et al.’s 2012 study and present in at least five case reports [1,6-9]. Several patients required orogastric feeding tubes or invasive gastrostomies for adequate nutrition [2,9]. Less commonly, two cases of GERD and one of pyloric stenosis have also been reported in children [1,6,9]. The only known adult case with GI manifestations, a 24-year-old female, experienced severe GERD and EOE and had a significant history of other atopic diseases [5].

For our patient, the initial workup prioritized common causes of progressive non-bilious emesis and nausea. Cholelithiasis and biliary dysfunction as a potential explanation for delayed gastric emptying were considered but excluded given the negative HIDA scan and abdominal ultrasound results. H. pylori-associated gastritis and peptic ulcer disease were ruled out via EGD with biopsy. Colonoscopy was deferred due to the absence of lower GI symptoms.

In the GI clinic, GERD was initially suspected due to its previously reported association with 10p15.3 microdeletion syndrome in both children and adults [1,5,7,8,10]. A repeat EGD with a 96-hour pH study ruled out pathologic GERD and EOE, the only other known GI pathology reported in adult patients with 10p15.3 microdeletion syndrome [5]. At this point, her abnormal gastric emptying scintigraphy and non-response to metoclopramide suggested possible refractory gastroparesis [11]. While gastroparesis is not a known phenotype of 10p15.3 microdeletion syndrome, other neurodevelopmental delay conditions such as Angelman syndrome and Prader-Willi syndrome have been associated with slowed gastric emptying, prompting us to explore this possibility in her case [12,13].

Her significant subsequent improvement on prucalopride, in the absence of other explanations for gastroparesis (e.g., diabetes, surgical history, hypothyroidism, opioid use), suggested a diagnosis of refractory atypical gastroparesis linked to 10p15.3 microdeletion syndrome - an association not previously documented. Notably, her symptoms of eructation and bloating align with the atypical gastroparesis phenotype described by Szeto et al., which differs from idiopathic, diabetic, or postsurgical gastroparesis, where symptoms of early satiety and postprandial fullness are more evident [14]. Normal EGD ruled out structural abnormalities as a cause, despite the prevalence of dysmorphic features in patients with this syndrome. This case highlights the importance of considering motility disorders in patients with 10p15.3 microdeletion syndrome presenting with unexplained GI symptoms and raises questions about the underlying mechanisms driving these findings.

Research on the pathophysiology of 10p15.3 microdeletion syndrome has focused on the roles of *ZMYND11* and *DIP2C* genes. *ZMYND11* encodes a transcriptional repressor involved in the regulation of brain development, while *DIP2C* is highly expressed in the nervous system and has been linked to neurocognitive deficits [3,6,15,16]. A recent analysis found evidence that loss-of-function of *ZMYND11* resulted in intellectual disability, hypotonia, dysmorphic features, and feeding difficulties [17]. Similar mechanisms of haploinsufficiency underlie other genetic disorders involving terminal deletions of chromosome 10p, such as HDR (Hypoparathyroidism, Deafness, and Renal dysplasia) syndrome and DiGeorge syndrome type 2 [18].

In the context of atypical gastroparesis, enteric nervous system dysfunction or vagus nerve impairment could provide an explanation for 10p15.3 microdeletion symptoms [19]. Recent studies have highlighted the role of autonomic dysfunction in the pathogenesis of gastroparesis, with evidence showing impaired vagus nerve activity leading to delayed gastric emptying [19]. Additionally, pilot studies on non-invasive vagal nerve stimulation, such as transcutaneous cervical vagal nerve stimulation, have demonstrated improvement in symptoms in patients with idiopathic and diabetic gastroparesis, suggesting a potential therapeutic mechanism [20]. This case of refractory atypical gastroparesis expands the known GI phenotype of 10p15.3 microdeletion syndrome and emphasizes the need for further investigation into these mechanistic pathways.

Additionally, this case underscores the importance of close follow-up and individualized management strategies for patients with rare genetic syndromes. Regular near-monthly visits allowed for timely therapy adjustments and provided patient reassurance, fostering treatment adherence despite chronic symptoms and understandable frustration. For rare disorders like 10p15.3 microdeletion syndrome, where literature and guidelines remain limited, personalized care plans and humanistic communication are essential.

## Conclusions

This case represents the first documented association between refractory atypical gastroparesis and 10p15.3 microdeletion syndrome in an adult patient. The patient's clinical course highlights the importance of a methodical diagnostic workup and the recognition of novel GI complications in rare genetic disorders, particularly where existing literature or guidelines are limited. Her significant improvement with prucalopride suggests a potential therapeutic avenue for other similar patients.

Notably, this case contributes to the evolving understanding of the GI phenotype in 10p15.3 microdeletion syndrome and emphasizes the need for further studies exploring the prevalence of motility disorders in this syndrome and its pathophysiological mechanisms, including enteric nervous system dysfunction and vagus nerve involvement. Clinicians should maintain a broad differential when evaluating unexplained GI symptoms in patients with rare genetic syndromes and provide close follow-up to optimize outcomes.
